# Gut microbiota modulation by plant polyphenols in koi carp (*Cyprinus carpio* L.)

**DOI:** 10.3389/fmicb.2022.977292

**Published:** 2022-10-12

**Authors:** Rong Zhang, Xin Kang, Lili Liu, Xiaowen Wang, Huijuan Li, Jianya Zhu, Yongchun Cao, Hua Zhu

**Affiliations:** ^1^Beijing Key Laboratory of Fishery Biotechnology, Fisheries Science Institute, Beijing Academy of Agriculture and Forestry Sciences, Beijing, China; ^2^Animal Science and Technology College, Beijing University of Agriculture, Beijing, China

**Keywords:** anti-inflammatory, anti-oxidative, anti-hyperglycemic, microbiome, ornamental fish, plant extracts

## Abstract

Plant polyphenol supplementation may improve fish health in aquaculture systems. To assess the potential benefits and function mechanism of plant polyphenols in aquaculture, fish were fed either basal feed (CON) or the basal feed supplemented with 500 mg/kg of curcumin (CUR), oligomeric proanthocyanidins (OPC), chlorogenic acid (CGA), or resveratrol (RES). After an 8-week feeding experiment, blood samples were used to analyze the concentrations of biochemical indices. Gut samples were collected to evaluate microbiota, short chain fatty acid (SCFA) levels, and gene expression. The results indicated that polyphenol administration reduced serum glucose and insulin. Lysozyme activity was enhanced by OPC and CGA, and superoxide dismutase activity was increased by CUR, OPC, and CGA. The gut microbial structure of the RES group was segregated from that of the CON, and the genus *Bacteroides* was identified as a potential biomarker in the CUR, CGA, and RES groups. Total gut SCFA increased in the CUR, CGA, and RES groups. A strong correlation was observed between *Bacteroides* and SCFA. In conclusion, dietary polyphenols have distinct anti-inflammatory, anti-oxidant, and anti-hyperglycemic activities that may be closely associated with their microbiota-modulation effects.

## Introduction

With the development of intensive aquaculture, outbreaks of aquatic diseases have become increasingly widespread. Antibiotics play a crucial role in the disease prevention and treatment. However, their abuse can cause a series of issues which have become challenges to the sustainable development of aquaculture (Limbu et al., 2021). As an alternative to antibiotics, plant polyphenols have extensive biological properties and could control diseases in a manner that is less risky in intensive aquaculture settings.

Plant polyphenols refer to a category of plant secondary metabolites that are widely found in nature and have phenolic hydroxyl structural units. CUR is a natural polyphenol derived from *Curcuma longa* Linn. (Moghadamtousi et al., 2014; Hewlings and Kalman, 2017). CGA is an ester of caffeic acid and quinic acid that is naturally found in green coffee extracts and tea (Naveed et al., 2018). RES belongs to the stilbenes, which are normally derived from grapes, peanuts, and knotweeds (Mohammad et al., 2009). OPC are a type of polyphenols formed from the condensation of catechins or epicatechins (Brillouet et al., 2017). They have been reported to exhibit a wide variety of biological and pharmacological activities, including antioxidant, anti-inflammatory, and antimicrobial effects (Gowd et al., 2019).

The gut microbial ecosystem plays a critical role in the digestion of nutrients and maintenance of health status in animals (Clements et al., 2009; Legrand et al., 2020; Sparagon et al., 2022). The host provides the living environment and nutrients for the gut flora, and gut bacteria participate in host nutrition and immune function (Pérez et al., 2010). Gut bacterial metabolites are also actively involved in host immunity modulation (Shibata et al., 2017). Plant polyphenols are present in the digestive tract in the form of bound phenols and free phenols, among which free polyphenols account for only 5–10% of the total polyphenols, while bound phenols account for 90–95% (Westfall and Pasinetti, 2019). The majority of polyphenols undergo extensive biotransformation mediated by the gut microbes and demonstrate significant prebiotic effects (Swanson et al., 2020). Gowd et al. (2019) reported that polyphenols increased the short chain fatty acids (SCFA) synthesis in the intestine, indicating their roles as potential mediators involved in gut immune function. SCFA mediate the transmission of information between the gut flora and the host immune system, in addition to playing important roles in host nutritional metabolism and immune regulation (Maslowski and Mackay, 2010). SCFA can bind G protein-coupled-receptors (GPCR) and restrain histone deacetylases, thus activating signal cascade reactions in immune regulation (Koh et al., 2016).

Based on these earlier findings, it can be hypothesized that the biological and pharmacological effects of plant polyphenols on the host may be closely related to gut bacteria and the concentration of SCFA. The mechanism may operate by altering the relative abundance of SCFA-producing bacteria, hence increasing the SCFA contents in the intestine. However, in Koi carp, an important ornamental fish with high economic value, this hypothesis has not been explored (Zhang et al., 2020). We, therefore, aimed to test the pharmacological effects of plant polyphenols and their influence on gut microbiota and SCFA in this fish species.

## Materials and methods

Fish were obtained from a commercial hatchery and acclimated them to the experimental rearing systems for 4 weeks before the feeding experiment. At the beginning of the experiment, a total of 135 fish (weighing an average of 15.2 ± 2.88 g) were allocated to five feeding groups: a control group fed a basal diet (CON), and treatment groups fed basal diets supplemented with 500 mg/kg of curcumin (CUR, >95 g/100 g purity, extracted from *C. longa* Linn.), oligomeric proanthocyanidins (OPC, >95 g/100 g purity, extracted from grape seeds), chlorogenic acid (CGA, >98 g/100 g purity, extracted from eucommia ulmoides oliver), and resveratrol (RES, >98 g/100 g purity, chemical synthesis). The plant polyphenols were purchased from Beijing Solarbio Science & Technology Co., Ltd. There were three replicates for each feeding group with nine fish per replicate. The fish were reared in three 200-L tanks for each feeding group (one tank per replicate) in a recirculating aquaculture system for 8 weeks. Fish were fed daily at 09:00 and 18:00, and the feeding amount was 2% of the average initial body weight. The inclusion rate of ingredients of the basal feed are listed in Table 1. During the experiment, the water volume was exchanged daily to maintain high water quality. The water temperature was maintained at 21 ± 1°C. The dissolved oxygen (DO) content was measured by a multiparameter water quality probe (AP-2000, Aquaread), and the values ranged from 8.0–8.8 mg/l. The concentrations of ammonium nitrogen and nitrite nitrogen were measured *via* commercial kits by a portable colorimeter (WTW pHotoFlex^®^ pH, Xylem analytics), and the values were below 0.5 mg/l and 0.02 mg/l, respectively.

**Table 1:** Ingredients and nutrient profiles of the feed.

Ingredients	%
Soybean meal	35.00
Fish meal	17.38
Wheat flour	18.47
Wheat bran	15.00
Distillers dried grains with soluble	10.00
Fish oil	3.15
Vitamin premix^1^	0.50
Trace mineral premix^2^	0.50
**Nutrients**	
Dry matter	89.24
Crude protein	35.00
Crude fat	7.00
Crude ash	6.93
Neutral detergent fiber	11.13
Acid detergent fiber	4.46

1 Vitamin premix contained: vitamin A 2,700 IU/kg, vitamin D 75 IU/kg, vitamin E 65 IU/kg, vitamin B1 2.30 mg/kg, vitamin B2 6.80 mg/kg, vitamin B6 6.90 mg/kg, vitamin K 6.90 mg/kg, folic acid 2.65 mg/kg, biotin 0.50 mg, niacin 29.7 mg/kg, D-pantothenic acid 21.0 mg/kg.

2 Trace mineral premix contained: Cu 5.6 mg/kg, Fe 75 mg/kg, Zn 100 mg/kg, Mn 10 mg/kg, I 1.15 mg/kg, and Se 0.2 mg/kg.

Following the 8-week rearing period, food was withheld for 24 h, and 9 fish were randomly chosen from each replicate and anaesthetized with 0.1 g/l of tricaine methane sulfonate. Caudal vein blood was sampled from 3 fish in each replicate, centrifuged at 10, 000 ×*g* for 8 min, and the serum was decanted to analyze the concentrations of lysozyme, superoxide dismutase (SOD), alkaline phosphatase (ALP), glucose, and insulin (*n* = 9 per treatment). Then, gut samples were collected from 9 fish in each replicate. The entire gut tracts were carefully removed through a ventral incision made in the fish. The first segments (0.5 cm) were sampled at approximately 3 cm of the gut anterior end to determine the mRNA expression of tumor necrosis factor α (tnf-α), interleukin-8 (il-8) and interleukin-10 (il-10). Then, the following second segments (2 cm) were sampled to collect contents for the assessment of SCFA, and the third segments (2 cm) were sampled to collect contents for the assessment of bacterial community. The gut content samples of 3 fish from the same replicate were pooled to ensure the representativeness of the samples (*n* = 9 per treatment). The volume of pooled contents from 3 fish in each replicate was approximate 0.1–0.2 ml. All the samples were frozen immediately in liquid nitrogen and then stored at −80°C until further analysis.

## Measurements

### Serum parameters

The lysozyme activity was determined by measuring the reduction in turbidity after bacterial lysis (HY-60061 kit), the SOD activity was determined by the pyrogallol autoxidation method (HY-60001 kit), and insulin was measured by enzyme linked immunosorbent assay (HY-D0001; Beijing Sino-uk Institute of Biological Technology, Beijing, China). Glucose was measured by the glucose oxidase method (HY-N002; BioSino Bio-Technology and Science Inc., Beijing, China).

### Quantitative real-time polymerase chain reaction

The total RNA was extracted using the RNAprep Pure Tissue Kit (DP431, Tiangen, Beijing, China). Equal amounts of RNA extracted from three fish from the same replicate were pooled, and cDNA synthesis was carried out using the FastKing gDNA Dispelling RT SuperMix (KR118, Tiangen, Beijing, China). TIANGEN Talent qPCR PreMix (FP209, Tiangen, Beijing, China) was used for the qRT-PCR. The 25-μL reaction solution was as follows: 12.5 μl 2 × SYBR qPCR Mix, 0.5 μl of forward and reverse primers, 1 μl cDNA template, and 10.5 μl RNase-free ddH2O. The PCR reaction was performed as follows: 95°C for 2 min; and 40 cycles of 95°C for 15 s, 60°C for 20 s, and 72°C for 30 s. Each assay was run in triplicate. The relative gene expression levels were calculated using the 2^–ΔΔ^CT method (Pfaffl, 2001). The primer sets for each gene are shown in Table 2.

**Table 2:** Primer sequences.

Gene name	Primer sequences
β-actin	F: AGACATCAGGGTGTCATGGTTGGT
	R: CTCAAACATGATCTGTGTCAT
TNF-α	F: ACAGGTGATGGTGTCGAGGAGGA
	R: TCTGAGACTTGTTGAGCGTGAAG
IL-8	F: CTGCTGGTGTTTTGTTGG
	R: GAGTCTTAGAGGTCTGGGTG
IL-10	F: AGTCCTTATGGCTGTCACG
	R: TTTCAGTATATCCCGCTTG

### Short chain fatty acids

An aliquot of 50 mg gut contents was mixed with 50 μl of 15% phosphoric acid, 100 μl of 125 μg/ml isohexanoic acid, and 400 μl of diethyl ether. Samples were homogenated and then centrifuged at 12,000 ×*g* for 10 min at 4°C. Supernatants were analyzed using gas chromatography-mass spectrometry (GC-MS). The operation conditions were as follows: an Agilent HP-INNOWAX column, 30 m × 0.25 mm × 0.25 μm; carrier gas, helium; gas flow, 1.0 ml/min; split ratio, 10:1; injection volume, 1.0 μl; injector temperature, 250°C; ion source temperature, 230°C; interface temperature, 250°C; and quadrupole temperature, 250°C. The detector was operated in electron impact ionization mode (electron energy 70 eV) using full scanning and selected ion monitoring mode. Standards curves for acetate, propionate, butyrate, isobutyrate, valerate, isovalerate, and caprioate were used to quantify the SCFA in gut samples.

### Gut bacterial community

The total DNA was extracted using the TIANamp Stool DNA kit (Tiangen, Beijing, China). The 341f/806r primer set (341F: 5′-CCTAYGGGRBGCASCAG-3′; 806R: 5′-GGACTACNNGGGTATCTAAT-3′) was used to amplify the V3–V4 region of the 16S rRNA gene. TruSeq^®^ DNA PCR-Free sample preparation kit was used to construct sequencing libraries (Illumina, United States), and sequencing was performed using an NovaSeq6000 platform (Novogene Bioinformatics Technology Company, Tianjin, China).

Paired-end reads were merged using FLASH (v1.2.7; Magoč and Steven, 2011). Quality filtering was performed to produce high quality clean reads from raw reads according to QIME (v1.9.1) quality control process (Caporaso et al., 2010). UCHIME was used to remove chimeric sequences (Edgar et al., 2011). UPARSE (v7.0.1001) was used to cluster sequences into operational taxonomic units (OTUs) based on 97% similarity (Edgar, 2013). Taxonomic classifications were assigned using the SILVA SSURef database and RDP classifier *via* QIIME (Quast et al., 2012). Read depths were normalized to a depth of 58,548 reads/sample for all samples. In addition, the alpha diversity as indicated by the Chao1, ACE, Shannon, and Simpson indices were calculated, and a principal coordinate analysis (PCoA) based on weighted UniFrac distance was conducted using QIIME. A linear discriminant analysis of the effect size (LefSe) was performed at each rank of classification to estimate differentially represented bacteria using a logarithmic linear discriminant analysis (LDA) threshold score set to 4.0 (Segata et al., 2011).

### Statistical analysis

Differences in serum parameters, the concentration of SCFA, and mRNA expression between the polyphenol treatment groups and control groups were assessed using a one-way analysis of variance in SAS (SAS Institute, Cary, NC, United States). Chao1, ACE, Shannon, and Simpson indices were analyzed by *t*-tests to detect differences between the control and polyphenol-treated groups. An analysis of similarity randomization test (ANOSIM) was conducted using anosim function in vegan package in R (v.2.15.3) to identify differences in the gut microbiota structure. Statistical significance was determined at *p* < 0.05. A Kruskal-Wallis test was used for LefSe, and the logarithmic LDA score was 4.0. Non-parametric spearman rank correlation analysis was performed by the PROC CORR procedure of SAS (SAS Institute, Inc., Cary, NC, United States).

## Results

### Serum parameters

**Figure 1:**
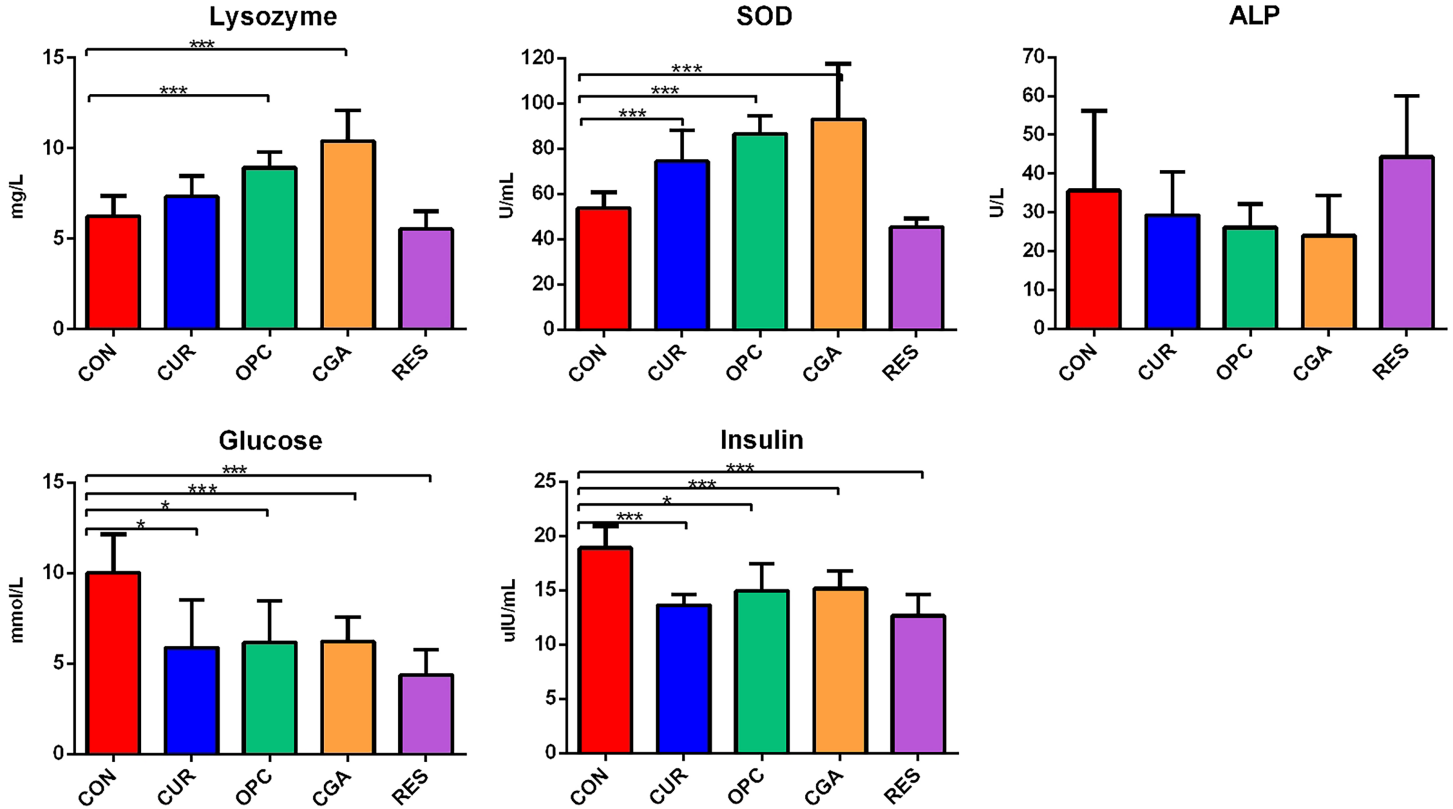
Effect of plant polyphenols on blood parameters of fish (*n* = 9 per treatment). Data are presented as mean ± standard deviation. Significant differences were labeled as * and *** for *p* < 0.05 and *p* < 0.001, respectively. SOD, Superoxide dismutase, ALP, alkaline phosphatase, CON, control group; CUR, curcumin group; OPC, oligomeric proantho cyanidins group; CGA, chlorogenic acid group; RES, resveratrol group.

OPC or CGA administration significantly elevated the lysozyme activities (*p* < 0.0001, *p* < 0.0001). SOD activity was significantly increased in the CUR, OPC, and CGA groups compared to the control (*p* < 0.0009, *p* < 0.0001, and *p* = 0.0003, respectively). The oral administration of CUR, OPC, CGA, and RES decreased the concentrations of glucose (*p* = 0.0130, *p* = 0.0128, *p* = 0.0040, and *p* = 0.0003, respectively) and insulin (*p* = 0.0002, *p* = 0.0132, *p* = 0.0054, *p* = 0.0003). There was no difference in the ALP activity between that in the control and the CUR, OPC, CGA, and RES treatment groups (Figure 1).

### Gut gene expression

**Figure 2:**
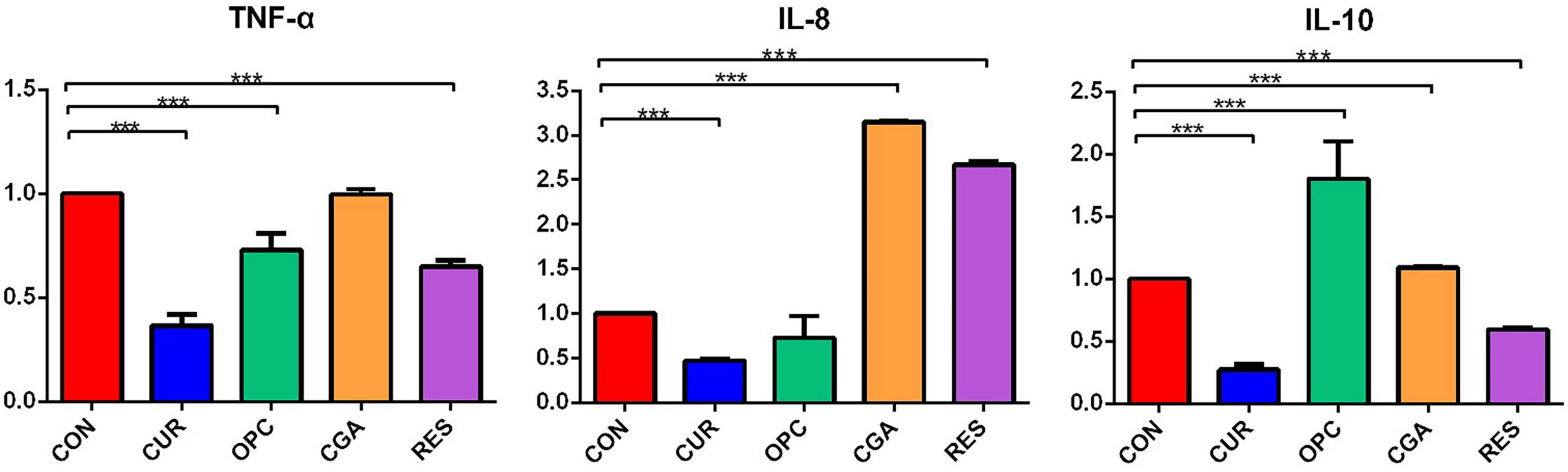
Relative expression of *tnf-α*, *il-8* and *il-10* genes in response to dietary plant polyphenols (*n* = 9 per treatment). Data are presented as mean ± standard deviation. Significant differences were labeled as *** for *p* < 0.001. CON, control group; CUR, curcumin group; OPC, oligomeric proantho cyanidins group; CGA, chlorogenic acid group; RES, resveratrol group.

The mRNA expression of *tnf-α* was significantly decreased in the CUR, OPC, and RES groups (*p* < 0.0001, *p* = 0.0042, and *p* < 0.0001, respectively). The mRNA expression of *il-8* was significantly decreased in the CUR group (*p* < 0.0001), but significantly increased in the CGA and RES groups (*p* < 0.0001, and *p* < 0.0001, respectively). The mRNA expression of *il-10* was significantly decreased in the CUR, CGA and RES groups (*p* < 0.0001, *p* = 0.0094, and *p* = 0.0001, respectively), but significantly increased in the OPC group (*p* < 0.0001; Figure 2).

### Short chain fatty acids

**Figure 3:**
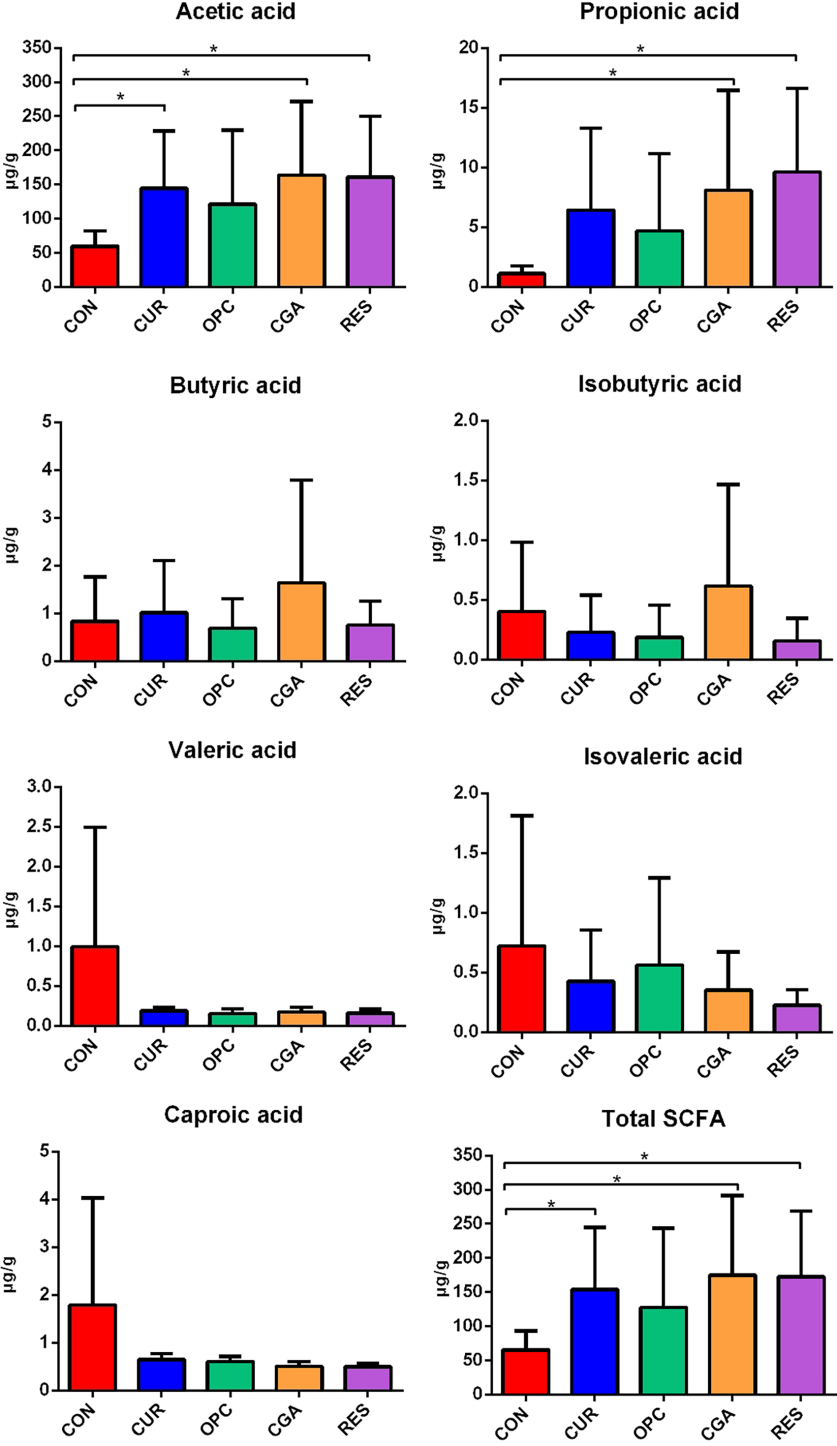
Effect of plant polyphenols on the concentration of short chain fatty acids (*n* = 9 per treatment). Data are presented as mean ± standard deviation. Significant differences were labeled as * for *p* < 0.05. CON, control group; CUR, curcumin group; OPC, oligomeric proantho cyanidins group; CGA, chlorogenic acid group; RES, resveratrol group.

Dietary supplementation with CUR, CGA, and RES significantly increased the acetic acid level in the gut (*p* = 0.0232, *p* = 0.0285, and *p* = 0.0130, respectively). The oral administration of CGA and RES increased the propionic acid level (*p* = 0.0486, and *p* = 0.0074, respectively). Total SCFA levels were increased in the CUR, CGA, and RES groups (*p* = 0.0309, *p* = 0.0325, and *p* = 0.0158, respectively). However, there were no differences in the levels of butyric acid, isobutyric acid, valeric acid, isovaleric acid, and caproic acid between the control and all polyphenol-treated groups (Figure 3).

### Gut microbiota

**Figure 4:**
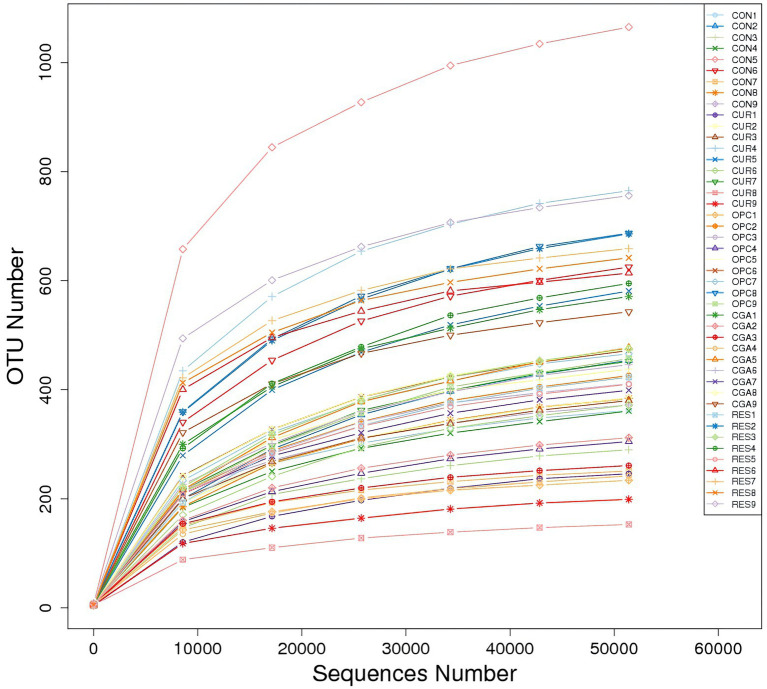
The rarefaction curves.

**Figure 5:**
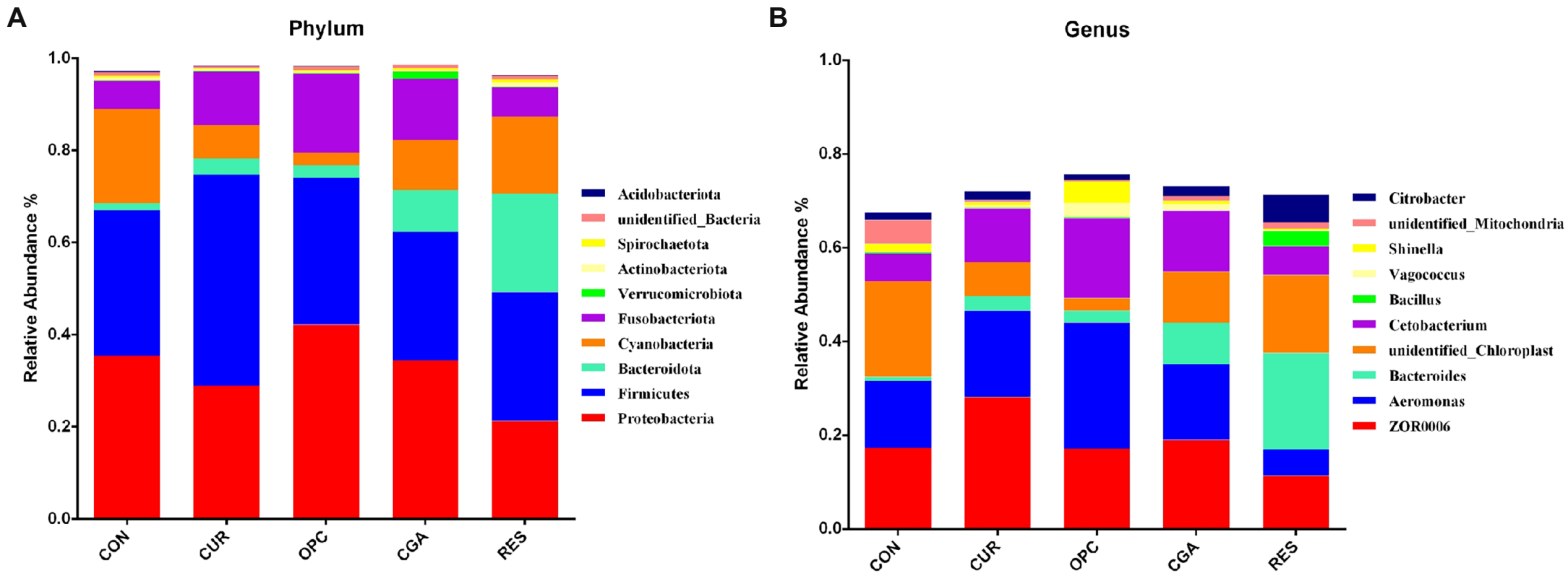
The relative abundance of gut bacteria at the level of **(A)** phylum and **(B)** genus (*n* = 9 per treatment). Only the top 10 bacteria were presented. CON, control group; CUR, curcumin group; OPC, oligomeric proantho cyanidins group; CGA, chlorogenic acid group; RES, resveratrol group.

A total of 3,929 bacterial operational taxonomic units (OTUs) were obtained using >97% nucleotide sequence identity. The rarefaction curves illustrated sufficient OTU coverage to describe the bacterial diversity accurately (Figure 4). The most predominant phylum in all samples was Firmicutes (33.07%), followed by Proteobacteria (32.44%), Cyanobacteria (11.58%), Fusobacteriota (10.88%), Bacteroidetes (7.64%), unidentified_Bacteria (0.70%), Actinobacteriota (0.62%), Verrucomicrobiota (0.37%), Spirochaetota (0.34%) and Acidobacteriota (0.12%). The genus *ZOR0006* (18.56%) dominated in all samples, followed by *Aeromonas* (16.28%), unidentified_Chloroplast (11.57%), *Cetobacterium* (10.71%), *Bacteroides* (7.20%), *Citrobacter* (2.54%), unidentified_Mitochondira (1.68%), *Shinella* (1.61%), *Vagococcus* (0.99%) and *Bacillus* (0.82%; Figure 5).

**Figure 6:**
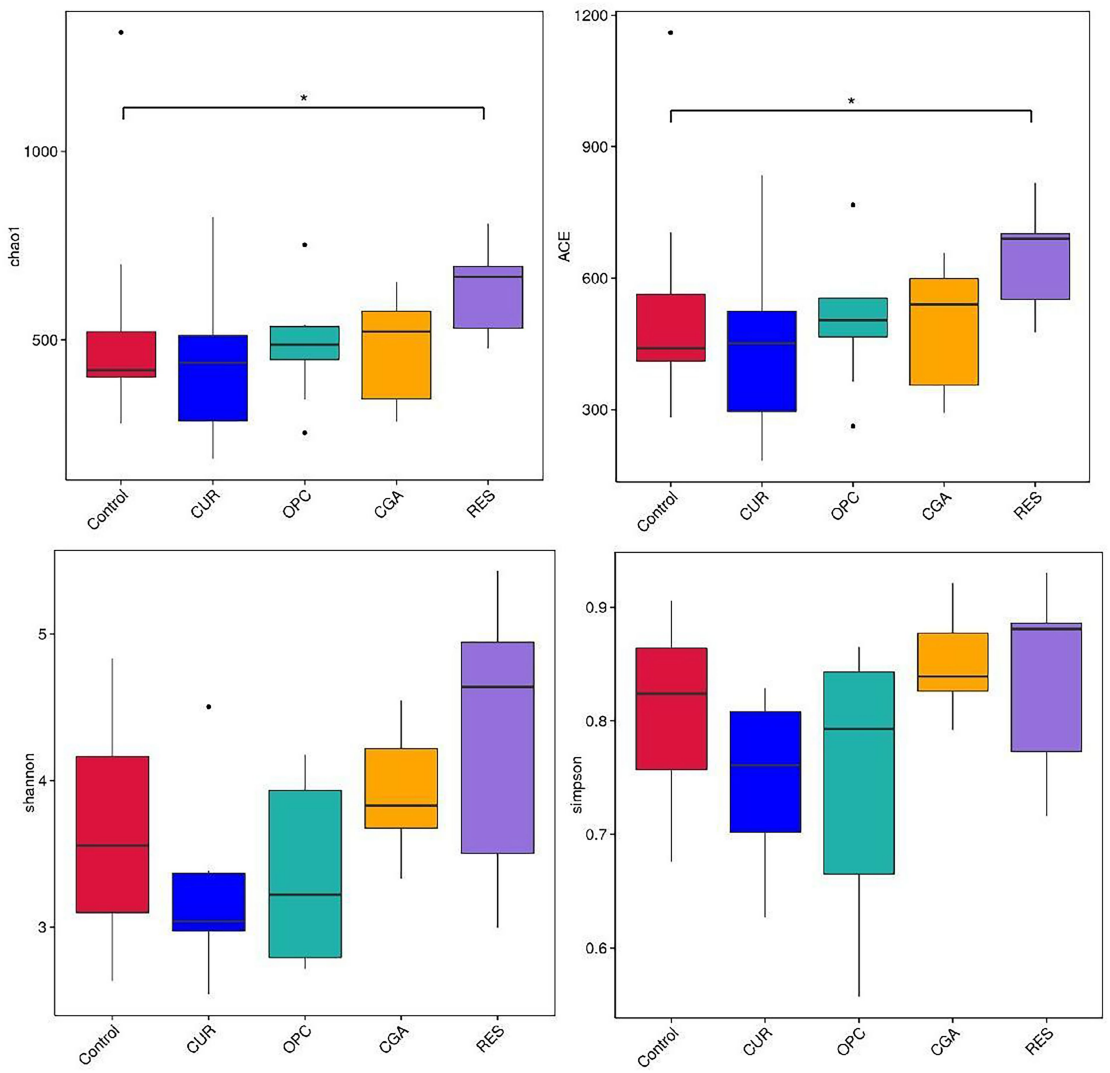
Alpha-diversity indices of gut bacterial community (*n* = 9 per treatment). CON, control group; CUR, curcumin group; OPC, oligomeric proantho cyanidins group; CGA, chlorogenic acid group; RES, resveratrol group.

**Figure 7:**
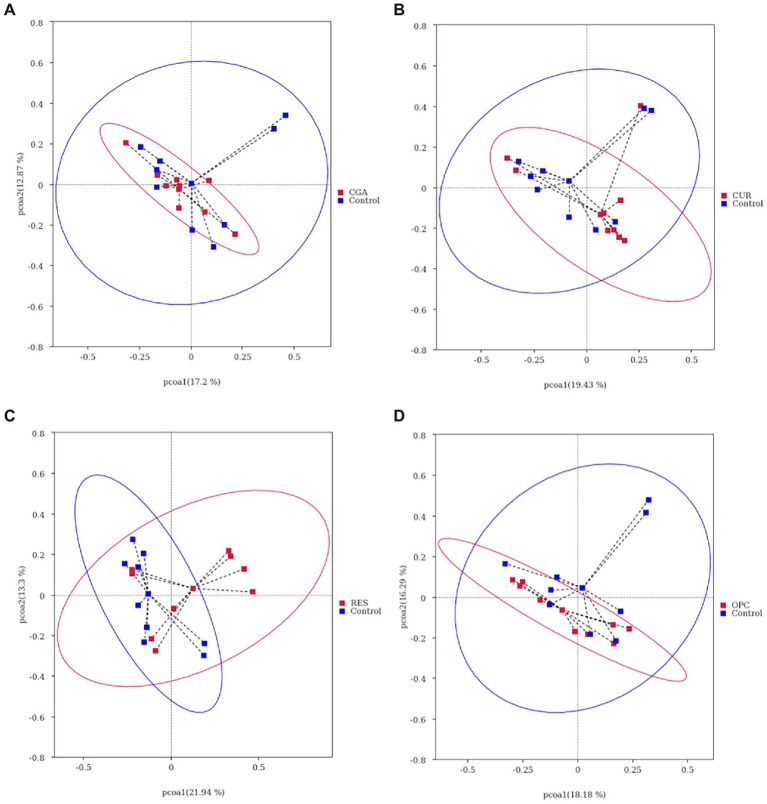
Gut bacterial community of fish between **(A)** CUR and control; **(B)** OPC and control; **(C)** CGA and control; **(D)** RES and control, differentiated using a principal coordinate analysis based on bray crutis distances (*n* = 9 per treatment). CON, control group; CUR, curcumin group; OPC, oligomeric proantho cyanidins group; CGA, chlorogenic acid group; RES, resveratrol group.

Dietary supplementation with RES increased the gut bacterial richness, as indicated by the Chao1 and ACE (*p* = 0.017 and *p* = 0.0257, respectively). However, no differences were observed in alpha-diversity between the control and other polyphenol-treated groups (Figure 6). The gut microbiota of the RES group was segregated from those of the control, as indicated by the PCoA based on Bray-Crutis distances and the analysis of the similarity randomization test (*R* = 0.2298, *p* = 0.032). No difference was detected in the bacterial community between the control and the CUR group (*R* = 0.05641, *p* = 0.166, ANOSIM), the control and the CGA group (*R* = 0.09122, *p* = 0.111, ANOSIM), or the control and the OPC group (*R* = 0.1456, *p* = 0.057, ANOSIM; Figure 7).

**Figure 8:**
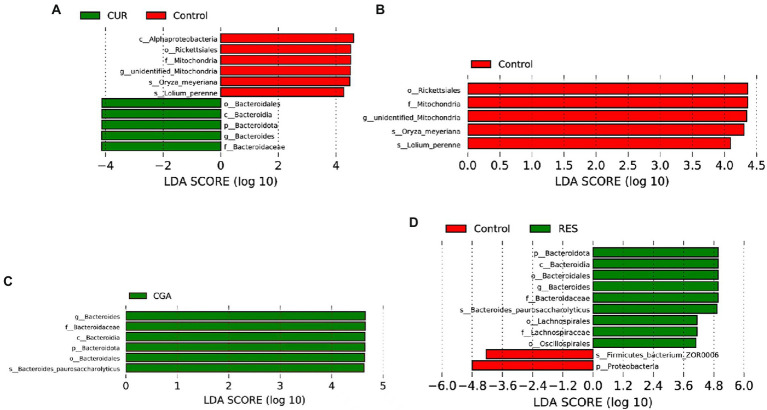
Differentially represented bacteria by LEfSe between **(A)** CUR and control; **(B)** OPC and control; **(C)** CGA and control; **(D)** RES and control (*n* = 9 per treatment). CON, control group; CUR, curcumin group; OPC, oligomeric proantho cyanidins group; CGA, chlorogenic acid group; RES, resveratrol group.

LefSe was performed to identify bacterial biomarkers that were differentially represented between the control and CUR, OPC, CGA, and RES groups, separately. The genus *Bacteroides* was identified as a potential biomarker in the CUR, CGA, and RES groups (*p* = 0.01517, *p* = 0.00411, *p* = 0.00035, respectively; Figure 8).

**Figure 9:**
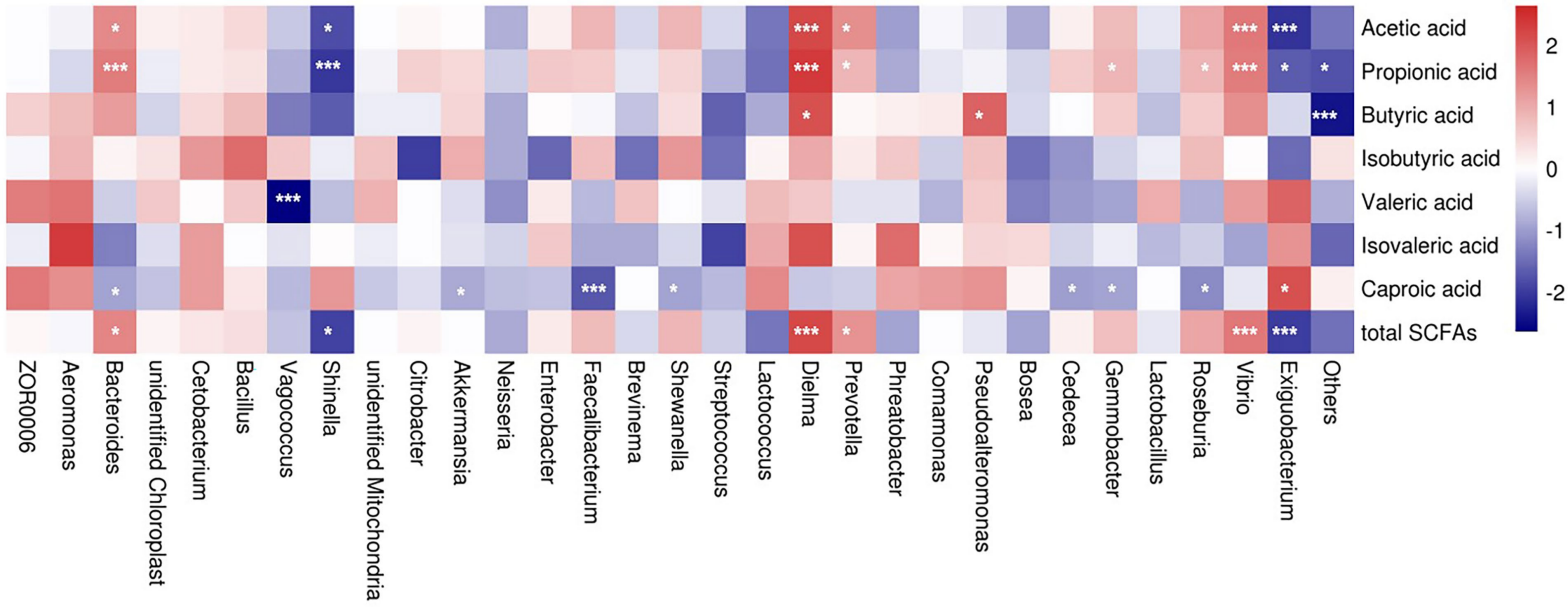
Correlation analysis between relative genus abundance and short chain fatty acids. Red denotes a positive correlation and blue denotes a negative correlation. Color intensity reflects the degree of correlation. Significant differences were labeled as * and *** for *p* < 0.05 and *p* < 0.001, respectively.

To further identify a possible relationship between SCFA level and bacterial abundance at the genus level, we conducted a non-parametric spearman rank correlation analysis of all five groups. The results revealed that genus *Bacteroides* abundance was positively related to the concentrations of acetic acid (*p* = 0.0149), propionic acid (*p* = 0.0008), and total SCFA (*p* = 0.0119), whereas it was negatively correlated to the concentration of caproic acid (*p* = 0.0401; Figure 9).

## Discussion

Antibiotic resistance has emerged as a possible threat to human health, increasing public concern about the use of antibiotics in aquaculture. Polyphenols are a broad category of plant secondary metabolites that are composed of phenolic hydroxyl groups (Vinson et al., 2001). Flavonoids, phenolic acids, stilbenes, and lignans are the four major chemical families of dietary polyphenols (Thouvenot et al., 2022). These plant polyphenols have been reported to exhibit a wide variety of biological and pharmacological properties, such as antioxidant, anti-inflammatory, and anti-bacterial effects (Gowd et al., 2019). Therefore, they are considered promising substitutes for antibiotics due to their safety and appropriateness for use in aquatic feeds. Here, we sought to test the potential benefits of polyphenols supplementation for improving koi health.

Oxidative stress has been implicated in pathological conditions in fish disease (Welker and Congleton, 2005). Oxidative stress occurs when the reactive oxygen species (ROS) production exceeds the ability of the anti-oxidative system to remove ROS (Ece et al., 2006). Various defense approaches, including non-enzymatic molecules and enzymatic scavengers of ROS, participate in combating ROS accumulation (Martindale and Holbrook, 2002). Polyphenols have at least one hydroxyl group capable of neutralizing free electrons, which contributes to their outstanding antioxidant properties. CGA protects the body from oxidative stress by scavenging ROS and up-regulating the activities of SOD, catalase, and glutathione peroxidase (Cha et al., 2014; Hao et al., 2015). The antioxidative activity of CUR has been attributed to the presence of methoxy, phenoxy, and carbon–carbon double bonds in its structure (Venugopal and Adleri, 2007). Mohammadi et al. (2021) suggested that grapeseed proanthocyanidin extract exhibited an antioxidant activity by increasing the antioxidant enzymes activities at a low concentration, while it functioned as a free radical scavenger at a high concentration. In aquatic animals, dietary supplementation with plant polyphenols greatly enhanced antioxidant activity and the expression of antioxidant genes but decreased malondialdehyde contents (Yonar et al., 2019; Zhang et al., 2019; Mousavi et al., 2020). Similar results were observed in this research in which serum SOD activity was significantly enhanced by dietary CUR, OPC, and CGA. RES regulates mitochondrial ROS homeostasis by activating the Sirt3 signaling pathway (Zhou et al., 2014). However, the serum SOD activity was not affected by dietary supplementation of RES at 500 mg/kg. A similar result was reported that the activities of catalase and SOD were not affected by RES administration at 300 mg/kg, but the activity of glutathione peroxidase increased significantly in common carp (Martina et al., 2022).

Plant polyphenols also exhibit distinct anti-inflammatory activity. Grapeseed proanthocyanidins had beneficial effects on the immune function of tilapia fingerlings by increasing serum lysozyme activity and albumin levels (Zhai et al., 2014). This is in accordance with our result that dietary OPC increased serum lysozyme and the expression of anti-inflammatory cytokines *il-10*, but decreased the expression of pro-inflammatory cytokines *tnf-α*. CUR reduces the stimulation of pro-inflammatory cytokines by inhibiting NF-κB or through direct binding with TNF-α (Zhou et al., 2011; Anthwal et al., 2014). This was also supported by the results of the present study, in which dietary CUR decreased the expression levels of *tnf-α* and *il-8* in the intestines. Similarly, the anti-inflammatory activity of CGA is associated with the regulation of NF-κB and toll-like receptor signaling pathways (Shi et al., 2013; Shin et al., 2015; Lou et al., 2016). RES modulates inflammatory reactions by improving the mRNA expression of anti-inflammatory cytokines and inhibiting that of pro-inflammatory cytokines in turbot (Castro et al., 2008; Tan et al., 2019). In a previous study, supplemental *Eucommia ulmoides* leaf powder, which is high in CGA, greatly enhanced the mRNA expression of inflammatory cytokines in *Scophthalmus maximus* L. (Zhang et al., 2019). In the present study, it was observed that the mRNA expression of inflammatory cytokines was enhanced in response to dietary CGA and RES. It is possible that gut metabolites, such as SCFA, might also participate in the regulation of immune gene expression. Li et al. (2022) reported that dietary supplementation with sodium acetate, sodium propionate, or sodium butyrate upregulated the mRNA expression of inflammatory cytokines. We observed that the concentrations of acetic acid, propionic acid, and total SCFA were enhanced by dietary CGA and RES, which may explain the up-regulated expression level of *il-8* in these two treatment groups.

A growing number of studies have been conducted on the interrelation between polyphenol intake and the lower risk of diabetes in humans (Kim et al., 2016). However, reports on the antihyperglycemic effects of polyphenol in fish nutrition are still scarce. Fish, especially carnivores, have low tolerance to feed starch mainly due to their low glucose uptake and slow clearance of blood glucose (Kamalam et al., 2017). Therefore, maintaining glucose homeostasis is of great importance to ensure the fish health. The results of this study revealed that polyphenols administration for 8 weeks significantly reduced the levels of serum glucose and insulin. Dietary polyphenols improved glucose homeostasis through a variety of mechanisms (Kim et al., 2016). The antihyperglycemic effects of polyphenols may be associated with the suppression of α-amylase, α-glucosidase (McDougall et al., 2005; Kwon et al., 2008), glucose uptake into brush border membrane vesicles of the intestine (Manzano and Williamson, 2010), and glucose release from the liver (Collins et al., 2007); the enhancement of pancreatic β-cell function (Coskun et al., 2005) and glucose uptake in the muscle and adipocytes (Pinent et al., 2004; Penumathsa et al., 2008). A meta-analysis indicated that RES intervention had a favorable effect on glucose control and insulin sensitivity in diabetic participants, but did not improve those indices in non-diabetic participants (Liu et al., 2014). In prediabetic patients, CUR improved insulin resistance and pancreatic β cell function (Chuengsamarn et al., 2012).

The biological activity of polyphenols may be closely related to the composition of SCFA-producing bacteria and SCFA content in the gut of fish. We observed that dietary supplementation with polyphenols significantly increased the relative abundance of genus *Bacteroides*, which was positively related to the concentration of acetic acid, propionic acid, and total SCFA. This was consistence with a previous study that the abundance of *Bacteroides* and other members of the phylum Bacteroidetes was strongly correlated to the concentration of SCFA in the gut of mice (Zhao et al., 2013). Similarly, Gowd et al. (2019) suggested that polyphenols increased SCFA synthesis in the intestine, and recognized SCFA as potential mediators involved in gut immune functions. SCFA mediate information transmission between the gut flora and the host immune system and play important roles in host nutritional metabolism and immune regulation (Maslowski and Mackay, 2010). The oral administration of sodium acetate and sodium propionate enhanced the non-specific immune responses and antioxidative capability of fish (Ehsan et al., 2002; Hoseinifar et al., 2016; Li et al., 2022). Additionally, the enhanced SCFA-producing bacteria and SCFA content in the gut may also contribute to the glucose homeostasis of fish. Acetate was effective in improving insulin sensitivity and regulating glucose-stimulated insulin secretion, possibly *via* the SCFA-specific GPCR (Yamashita et al., 2007; Tolhurst et al., 2012). Sung et al. (2017) reported that RES administration enhanced the *Bacteroides* abundance, and further fecal transfer from RES-fed mice ameliorated glucose homeostasis in obese mice, suggesting that RES-induced modifications in the gut microbiota were associated with improved glucose homeostasis.

In conclusion, dietary polyphenols have distinct anti-inflammatory, anti-oxidant, and anti-hyperglycemic activities that may be closely associated with their microbiota-modulation effects and the concentration of SCFA in the gut. Polyphenols are likely to provide a powerful means of increasing fish health in aquaculture, although further research is needed on additional species before polyphenols are applied more broadly in aquaculture.

## Data availability statement

The datasets presented in this study can be found in online repositories. The names of the repository/repositories and accession number(s) can be found at: https://www.ncbi.nlm.nih.gov/, PRJNA850142.

## Ethics statement

The animal study was reviewed and approved by the Animal Ethics Committee of the Fisheries Science Institute, Beijing Academy of Agriculture and Forestry Sciences.

## Author contributions

RZ and HZ: designed the study and drafted the manuscript. RZ, XK, LL, XW, HL, and JZ: performed the experiments. YC and HZ: revised the manuscript. All authors contributed to the article and approved the submitted version.

## Funding

This research was supported by the National Natural Science Foundation of China (32102801), Youth Research Fund of Beijing Academy of Agriculture and Forestry Sciences (QNJJ202246), and Beijing Innovation Consortium of Agriculture Research System, Beijing, China (BAIC07-2022).

## Conflict of interest

The authors declare that the research was conducted in the absence of any commercial or financial relationships that could be construed as a potential conflict of interest.

## Publisher’s note

All claims expressed in this article are solely those of the authors and do not necessarily represent those of their affiliated organizations, or those of the publisher, the editors and the reviewers. Any product that may be evaluated in this article, or claim that may be made by its manufacturer, is not guaranteed or endorsed by the publisher.
